# Various dimensions of sexual self‐concept in middle‐aged men after vasectomy: a matched analysis

**DOI:** 10.1111/bju.70351

**Published:** 2026-06-09

**Authors:** Lilly J. Schmalbrock, Camille Nöckel, Valentin H. Meissner, Matthias Jahnen, Stefan Schiele, Martina Kron, Helga S. Schulwitz, Andreas Dinkel, Jürgen E. Gschwend, Kathleen Herkommer

**Affiliations:** ^1^ Department of Urology, School of Medicine and Health, TUM University Hospital Technical University of Munich Munich Germany; ^2^ Department of Psychosomatic Medicine and Psychotherapy, School of Medicine and Health, TUM University Hospital Technical University of Munich Munich Germany; ^3^ Institute of Epidemiology and Medical Biometrics University of Ulm Ulm Germany

**Keywords:** male contraception, masculinity, toughness, body image, sexual self‐esteem, perceived pressure with regard to sexuality, reproductive health, gender norms, gender roles

## Abstract

**Objectives:**

To compare men with and without vasectomy across four dimensions of sexual self‐concept—toughness, body image, sexual self‐esteem, and perceived pressure with regard to sexuality—and to examine differences in sexual behaviour and psychosocial factors.

**Subjects and Methods:**

Data were derived from the Bavarian Men's Health Study, a cross‐sectional survey that collected detailed information on vasectomy status, sociodemographic and lifestyle factors, sexual behaviour, and psychosocial characteristics. Men with and without vasectomy were matched 1:1 using propensity score matching, adjusting for sociodemographic and lifestyle variables.

**Results:**

The final matched sample included 1140 men (mean [SD] age 50.6 [0.7] years). The mean (SD) time since vasectomy was 8.5 (5.7) years in the vasectomy group. There were no significant differences between men with and without vasectomy across any dimension of sexual self‐concept. Additionally, no significant group differences were observed in terms of symptoms of depression or anxiety. However, men who had undergone vasectomy reported significantly higher frequency of sexual activity, greater sexual satisfaction, and greater perceived importance of sexuality (all *P* < 0.05).

**Conclusion:**

This large, matched cross‐sectional study suggests that vasectomy is not associated with negative impacts on key aspects of men's sexual self‐concept, even after an average of 8 years since the procedure. Vasectomised men reported more active and satisfying sex lives and placed greater importance on sexuality. These findings may help clinicians to address persistent concerns among men considering vasectomy, particularly fears about its impact on the sexual self‐concept.

**Abbreviations**
BMH: Bavarian Men's Health (Study)
BMI: body mass index
EORTC QLQ‐C30: European Organisation for Research and Treatment of Cancer quality of life questionnaire‐30‐item core
GAD‐2: two‐item Generalized Anxiety Disorder questionnaire
PHQ‐2: two‐item Patient Health Questionnaire
QoL: quality of life
TUM: Technical University of Munich

## Introduction

### Background: Safety and Effectiveness of Vasectomy

Vasectomy is a safe and effective contraceptive procedure, particularly recommended for men who have completed their family planning [1]. With its simplicity, high success rates, and very low complication rates, vasectomy represents the most effective method of contraception for men, especially given the limited alternative options available for men [2]. Most vasectomies are performed in an outpatient setting and are well‐tolerated with local anaesthesia alone [1, 2]. A recent study from the UK, which analysed ~99 000 vasectomies performed over 15 years, concluded that the procedure is even safer than previously reported in national guidelines, with lower rates of postoperative complications such as haematomas (1.56%) and post‐vasectomy pain syndrome (0.14%) [3].

### Global Utilisation and Gender‐Related Barriers

Despite this, vasectomy rates remain low worldwide, with a further decline observed globally in the last two decades [4]. The rate of vasectomy usage, compared to other contraceptive methods like tubal ligation, can be seen as an indicator of gender inequality [2]. It is important to note that since the 1960s and the introduction of the birth control pill, contraception has been largely considered the responsibility of women, aligning with traditional gender roles. While these roles generally place more constraints on women's access to family planning and reproductive health services, men also face gender‐related barriers and are less frequently included in counselling on contraceptive options [2].

### Social, Cultural and Psychological Barriers

Commonly reported barriers to vasectomy include not only a lack of knowledge [2, 5], but also negative social, cultural, and moral norms or stigmas surrounding vasectomised men [6, 7]. Additionally, concerns persist that vasectomy may impair a man's sexual life, both physically (e.g., erectile problems) [8] and psychologically (e.g., as a perceived threat to traditional masculinity) [6, 7, 9]. Myths and misinformation further contribute to these perceptions by falsely equating vasectomy with castration and associating it with severe complications or personality changes [6, 7, 9]. However, research shows neither differences in erectile dysfunction rates between men with and without a vasectomy nor a negative impact on sexual satisfaction after vasectomy [8, 10, 11].

### Conceptual Background: Sexual Self‐Concept

Concerns about potential disturbances in the sexual self‐concept also play a significant role. The sexual self‐concept is broadly defined as an individual's perception and understanding of themselves as a sexual being. The sexual self‐concept develops through subjective interpretations of sexual experiences and external feedback from others, such as sexual socialisation and social comparisons [12]. However, there is no final consensus on its definition and operationalisation. Sexual self‐concept remains a weakly defined, broad construct. While some researchers focus on sexual aspects, others also include gender‐related domains [12].

### Dimensions of Sexual Self‐Concept Examined in the Present Study

In the present study, selected dimensions that have been examined in prior research on sexual self‐concept, namely body image and sexual self‐esteem were included [12]. Body image refers to individuals’ perceptions of their own body and their attitudes toward it, particularly in relation to sex life [13]. Sexual self‐esteem refers to a positive perspective on, and confidence in, one's ability to engage in satisfying and pleasurable sexual interactions with another person [14]. In addition to these established dimensions, we included exploratory domains that are conceptually related to sexual self‐concept but have been less frequently examined within this framework. Specifically, we assessed perceived pressure with regard to sexuality, referring to perceived external and self‐ascribed internal standards related to sexuality [15] and toughness, as an aspect of masculine norms that focuses on beliefs about traditional male behaviour [16]. Although these aspects have rarely been operationalised in quantitative assessments of the sexual self‐concept, theoretical perspectives such as the biopsychosocial model, as well as social and sexual script theories, suggest that internalised gender norms and perceived expectations regarding sexuality represent an important contextual basis shaping sexual self‐perceptions [17]. Because such norms influence the sexual identity of how individuals perceive and evaluate their sexuality and therefore the sexual self‐concept, these domains were included as exploratory sexual self‐concept‐related dimensions in the present study.

### Study Gap and Objective

Studies addressing the relationship between vasectomy and sexual self‐concept of men are limited and primarily focus on perceptions of vasectomy in relation to a man's sense of masculinity. These studies are mostly qualitative, based on interviews to explore opinions about vasectomy and the reasons for its low uptake [6]. Interestingly, while the fear of reduced manhood after vasectomy is quite well documented in qualitative research, studies investigating actual change in different dimensions of the self are sparse. In a 50‐year old small study with 20 men, no change in general self‐concept emerged from before to 18 months after vasectomy [18]. Since then, quantitative research has largely been lacking. Moreover, no study has comprehensively examined potential differences in the sexual self‐concept, understood as a multidimensional construct, between men with and without vasectomy. Therefore, the aim of this study was to compare men around the age of 50 years with and without a vasectomy concerning four dimensions of the sexual self‐concept.

## Subjects and Methods

### Study Design and Participants

Data collection was conducted as part of the Bavarian Men's HealthStudy (BMH‐Study) between 2020 and 2023 using questionnaires and physician interviews. A detailed description of the BMH‐Study was previously provided [19]. Men who responded to all questions regarding the four dimensions of sexual self‐concept, sexual behaviour and psychosocial variables were included. The study only included heterosexual men because previous research indicates that some aspects of the sexual self‐concept may differ significantly across sexual orientations [20, 21]. Exclusion criteria were vasovasostomy and/or bilateral orchidectomy. The study has been approved by the Ethics Committee of the Technical University of Munich (TUM) University Hospital (approval number: 69920SSR). All men were fully informed about the study procedure and provided written informed consent before their enrolment.

### Sociodemographic and Lifestyle Variables

Assessment of sociodemographic variables (such as partnership duration in months, presence of children, level of education, and self‐perceived economic situation) and lifestyle factors (including alcohol consumption, smoking status, body mass index [BMI], and physical activity) were assessed as described earlier in [19].

### Vasectomy‐Specific Variables

Vasectomy status (yes/no) was assessed during the physician's clinical interview. For those who had undergone a vasectomy, the time since the procedure and the age at which it was performed were recorded.

### Sexual Behaviour

#### Sexual Activity

Frequency of partnered sexual activity within the last 3 months was assessed with five answer options: ‘No’, ‘Once a month or less’, ‘A few times a month to once a week’, ‘Two to three times a week’, ‘Four times or more per week’. Additionally, the number of sexual partners across the lifespan was assessed with nine answer options (0, 1, 2–3, 4–5, 6–10, 11–15, 16–20, 21–30, >30) and then categorised into four groups (0–1, 2–10, 11–30, >30) [19, 22].

#### Satisfaction with Frequency of Sex

Participants’ satisfaction with the frequency of sex was determined by the question: ‘When you think about how often you have been sexually active in the last 3 months—would you wish for more or less sex?’ Response options included: ‘Wishing for more sex than actually had’, ‘Wishing for less sex than actually had’, and ‘Wishing it to remain the same as in the last 3 months’ [15, 19, 22].

#### Sexual Satisfaction and Perceived Importance of Sexuality

Sexual satisfaction was assessed using a single item: ‘How satisfied are you with your sex life?’ Respondents provided answers on a 5‐point Likert scale ranging from ‘very satisfied’, ‘satisfied’, ‘more or less satisfied’, ‘dissatisfied’ to ‘very dissatisfied’. The answers were then categorised in three groups: ‘very satisfied/satisfied’, ‘more or less satisfied’, ‘dissatisfied/very dissatisfied’. Perceived importance of sexuality was assessed using a single‐item: ‘How important is sexuality to you currently?’ Respondents provided answers on a 5‐point Likert scale ranging from ‘very unimportant’, ‘unimportant’, ‘more or less important’, ‘important’, and ‘very important’. The answers were then categorised into three groups ‘important’ (very important and important), ‘more or less important’ and ‘unimportant’ (very unimportant and unimportant) [19, 23].

#### Psychological Distress and Quality of Life (QoL)

Symptoms of depression and anxiety were assessed using the German version of the two‐question screening tools: Patient Health Questionnaire (PHQ‐2) and Generalized Anxiety Disorder (GAD‐2) [24]. Both measures are rated on a 4‐point Likert scale (0–3). A summary score ≥3 indicates clinical levels of depression or anxiety symptoms, respectively. These measures have proven reliable and valid in previous studies [24]. For the overall sample the inter‐item correlation was 0.52 for the PHQ‐2 and 0.46 for GAD‐2.

The QoL was assessed using the German version of items 29 and 30 from the European Organisation for Research and Treatment of Cancer quality of life questionnaire‐30‐item core (EORTC QLQ‐C30) [25, 26], a subscale designed to evaluate global health and overall QoL. Responses for these items were rated on a 7‐point Likert scale ranging from 1 (very poor) to 7 (excellent). Following the EORTC scoring guidelines, the two items were first summed to obtain a raw score ranging from 2 to 14. This score was then linearly transformed to a 0–100 scale using the formula ([s – 2]/12) × 100, where higher scores indicate better QoL. This transformation facilitates interpretability and comparability with other studies using the EORTC QLQ‐C30 scoring system. Based on reference values, a score of ≥70 is indicative of good QoL, whereas a score of <70 suggests poor QoL. Internal consistency (Cronbach's alpha) in the present sample was α = 0.83.

#### Dimensions of Sexual Self‐Concept

In this study four dimensions of the sexual self‐concept were assessed: toughness, body image, sexual self‐esteem and perceived pressure with regard to sexuality.

##### Toughness

To assess toughness, three items from the Toughness Norm subscale of the German version of the Male Role Norms Scale (MRNS) [16, 27] were used. The items were: ‘Nobody respects a man very much who frequently talks about his worries, fears, and problems’, ‘When a man is feeling a little pain, he should try not to let it show very much’, and ‘I think a young man should try to become physically tough, even if he's not big’. Responses to the questionnaire were rated on a 5‐point Likert scale (1 = fully disagree to 5 = fully agree). A higher score represents higher toughness and an understanding of a more traditional male role norm, namely the expectation that men should be tough and self‐reliant, whereas a lower score represents lower toughness. Cronbach's α was 0.67.

##### Body Image

Body image was assessed using three items from the 35‐item Dresden Body Image Inventory (DKB‐35) [13], potentially related to sexuality. The items were: ‘I like my body’, ‘I enjoy showing my body’ and ‘I have pleasant and intense feelings of my body in sexuality’. Participants rated each item on a 5‐point Likert scale, (1 = not at all to 5 = completely). Higher scores indicate a more positive body image. Cronbach's α was 0.67.

##### Sexual Self‐Esteem

Sexual self‐esteem was assessed using three items [28]: ‘During sex, I am a good lover’, ‘During sex, I pay attention to my partner's desires’, and ‘During sex, I am imaginative’. The participants rated each item on a 5‐point Likert scale (1 = not at all to 5 = completely). Higher scores represent higher sexual self‐esteem. Cronbach's α was 0.69.

##### Perceived Pressure with Regard to Sexuality

Four items measured the perceived pressure with regard to sexuality [15]: ‘I have the impression that nowadays, too much is expected from men during sex’, ‘With regards to sexuality, I feel put under pressure’, ‘I often panic about living up to the expectations of being a man during sex’, ‘I worry whether I correspond to the public image of a real man’. The response options ranged from 1 (not at all) to 5 (completely), with higher scores indicating greater perceived pressure with regard to sexuality. Cronbach's α was 0.76.

### Statistical Analysis

Descriptive statistics were used to outline the characteristics of the study population. Univariable comparisons between the vasectomy and non‐vasectomy groups were conducted to assess differences in sociodemographic and lifestyle factors using the chi‐square test. To ensure comparability between the groups, a 1:1 propensity score matching was conducted. Logistic regression was used to estimate the propensity score, incorporating variables such as sociodemographic variables (such as partnership duration in months, presence of children, level of education, and subjective economic situation) and lifestyle factors (including smoking status, BMI, and physical activity). The matching used a Greedy Nearest‐Neighbour algorithm with a 0.2 SD calliper width and exact matching for categorical variables. Post‐matching balance was evaluated using standardised differences. After successful matching the groups were compared regarding sexual behaviour, psychosocial variables and four dimensions of the sexual self‐concept using chi‐square test and Wilcoxon–Mann–Whitney test. All statistical tests were two‐sided, explorative and conducted at a significance level of 0.05. Moreover, effect sizes (Cramer's *V* and *r*) were computed. Data analyses were performed using the Statistical Analysis System (SAS), version 9.4 (SAS Institute Inc., Cary, NC, USA).

## Results

### Study Sample and Participant Exclusions

**Fig. 1:**
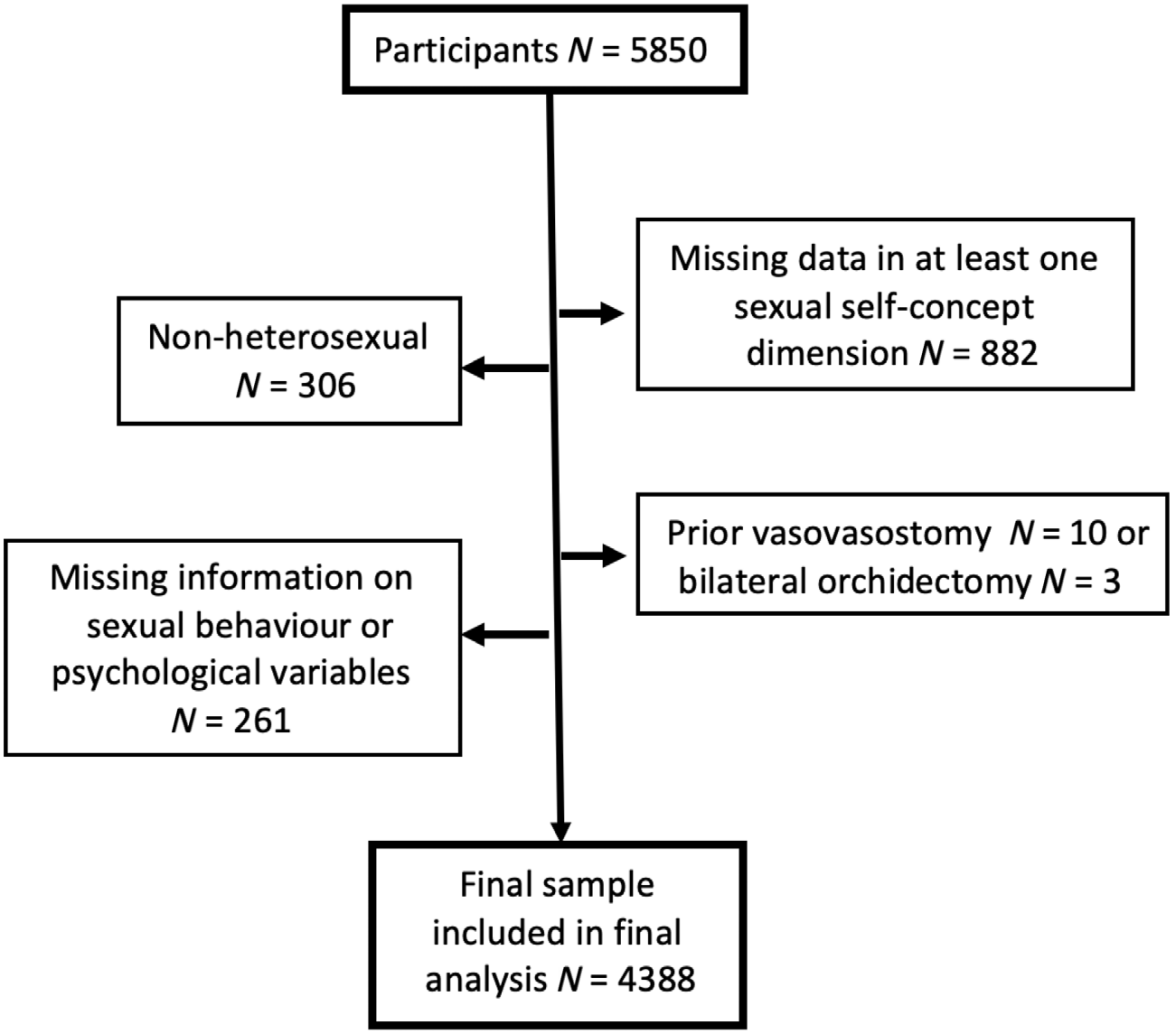
Flowchart of sampling procedure and exclusion criteria. This figure shows how the final sample for this analysis was selected.

Out of 5850 participants, 882 were excluded due to missing data in at least one sexual self‐concept dimension, leaving 4968 participants. Of these, 306 non‐heterosexual participants were excluded (leaving 4662). Another 10 were excluded due to prior vasovasostomy (leaving 4652), and three due to bilateral orchidectomy (leaving 4649). Additionally, 261 participants were excluded due to missing information on sexual behaviour or psychological variables (Fig. 1).

The final sample comprised 4388 participants with a mean (SD) age of 50.6 (0.7) years. Most participants (90.2%) were living in a stable partnership and had children (75%). Around three‐quarters (71.2%) reported a high level of education, and 85.7% reported a good or very good subjective economic situation (Table 1). Overall, 65.1% reported engaging in partnered sexual activity more than once per month, 63.3% considered their sex life as very important or important, and 46.0% were very satisfied or satisfied with it.

**Table 1:** Sociodemographic and lifestyle characteristics for the entire study sample (*n* = 4388) and comparison between the vasectomy (*n* = 595) and the no vasectomy groups (*n* = 3793).

Characteristic	Entire sample (*n* = 4388)	Vasectomy (*n* = 595)	No vasectomy (*n* = 3793)	*P*	Effect size
Vasectomy specific					
Time since vasectomy, years, mean (SD)		8.5 (5.7)			
Age at vasectomy, years, mean (SD)		42.2 (5.6)			
Sociodemographic factors					
Age, years, mean (SD)	50.6 (0.7)	50.6 (0.7)	50.6 (0.7)		
Partnership, % (*n*)					
Yes	90.2 (3957)	95.6 (569)	89.4 (3338)	**<0.001**	0.07
No	9.8 (428)	4.4 (26)	10.6 (402)		
Living together with partner, % (*n*)					
Yes	86.7 (3662)	90.1 (530)	86.2 (3132)	**0.009**	0.04
No	13.3 (560)	9.8 (58)	13.8 (502)		
Duration of partnership (years), % (*n*)					
0	9.9 (428)	4.4 (26)	10.7 (402)	**<0.001**	0.09
>0–5	8.4 (367)	10.2 (60)	8.2 (307)		
>5–10	8.3 (359)	10.2 (60)	8.0 (299)		
>10–20	29.9 (1296)	26.4 (156)	30.4 (1140)		
>20	43.6 (1891)	48.9 (289)	42.7 (1602)		
Children, % (*n*)					
Yes	75.0 (3290)	89.7 (534)	72.7 (2756)	**<0.001**	0.14
No	25.0 (1098)	10.3 (61)	27.3 (1037)		
Level of education*, % (*n*)					
Low	9.3 (408)	11.8 (70)	8.9 (338)	**<0.001**	0.07
Intermediate	19.5 (854)	24.5 (146)	18.7 (708)		
High	71.2 (3126)	63.7 (379)	72.4 (2747)		
Employment status					
Full‐time employed	92.7 (4060)	94.6 (562)	92.4 (3498)	0.122	0.04
Part‐time employed	4.9 (214)	4.2 (25)	5.0 (189)		
Unemployed (seeking work)	1.1 (49)	0.3 (2)	1.2 (47)		
Not employed (not seeking work)	1.3 (57)	0.8 (5)	1.4 (52)		
Subjective economic situation, % (*n*)					
(Very) good	85.7 (3752)	88.8 (526)	85.2 (3226)	**0.032**	0.04
Satisfactory	12.7 (556)	10.5 (62)	13.1 (494)		
Less good/poor	1.6 (68)	0.7 (4)	1.7 (64)		
Lifestyle factors					
Smoking, % (*n*)					
Yes (current smoker)	13.1 (571)	11.7 (69)	13.3 (502)	0.267	0.02
No (non‐/former‐smoker)	86.9 (3792)	88.3 (523)	86.7 (3269)		
Alcohol consumption, % (*n*)					
Low	16.4 (705)	12.9 (75)	17.0 (630)	**0.007**	0.04
Moderate	78.4 (3363)	83.3 (485)	77.6 (2878)		
Excessive	5.2 (223)	3.8 (22)	5.4 (201)		
Physical activity, % (*n*)					
≤1 time/week	13.3 (581)	12.6 (75)	13.4 (506)	**0.030**	0.04
2–5 times/week	61.6 (2692)	57.9 (344)	62.2 (2348)		
≥6 times/week	25.1 (1096)	29.5 (175)	24.4 (921)		
Waist circumference, cm, mean (SD)	97.1 (11.8)	96.9 (11.8)	97.1 (11.8)		
Waist circumference (cm), % (*n*)					
≤94	45.3 (1969)	45.5 (269)	45.3 (1700)	0.980	<0.01
>94–≤102	26.9 (1169)	26.6 (157)	27.0 (1012)		
>102	27.8 (1207)	27.9 (162)	27.8 (1042)		
BMI, kg/m^2^, mean (SD)	26.8 (4.2)	26.8 (4.1)	26.8 (4.2)		
BMI (kg/m^2^), % (*n*)					
<25	36.3 (1584)	34.2 (203)	36.6 (1381)	0.344	0.02
25–<30	45.9 (2003)	48.6 (289)	45.5 (1714)		
≥30	17.8 (776)	17.2 (102)	17.9 (674)		

Bold values statistically significant at *P* <0.05.

* Level of education: low (no secondary school certificate or lower secondary school certificate), intermediate (intermediate secondary school certificate), high (university entrance qualification or tertiary degree)

### Characteristics of Vasectomised and Non‐Vasectomised Men

Of the final 4388 participants, 595 (13.6%) were vasectomised and 3793 (86.4%) were not. The vasectomies had been performed at a mean (SD) of 8.5 (5.7) years prior and the men had a mean (SD) age of 42.2 (5.6) years. The rate of men who were living in a partnership was higher among men with vasectomy (95.6% vs 89.4%), and they reported more often to have children (89.7% vs 72.7%). Moreover, they were more likely to live together with their partner and report a partnership duration of >20 years (all *P* < 0.05). Vasectomised men also reported less often about excessive alcohol consumption compared to non‐vasectomised men (3.8% vs 5.4%, *P* = 0.007) and more often physical activity ≥6 times/week (29.5% vs 24.4%, *P* = 0.030). They rated their economic situation as (good or very good more often; 88.8% vs 85.2%), but their level of education was less often high (63.7% vs 72.4%) (all *P* < 0.05). There were no significant differences between groups regarding age, smoking status, BMI, waist circumference, or employment status (*P* > 0.05) (Table 1).

### Sexual Behaviour and Satisfaction After Propensity Score Matching

After successful propensity score matching the groups showed nearly zero differences in the matched parameters (sociodemographic and lifestyle, see Methods section) because we performed exact matching for all categorical variables. The standardised mean difference for BMI after matching was 0.015 kg/m^2^. Vasectomised men were significantly more likely to be sexually active with their partner within the last 3 months (*P* < 0.001; at least once per month: 77.9% vs 67.5%). Compared to non‐vasectomised men, vasectomised men more frequently rated their sexual satisfaction as satisfied or very satisfied (56.3% vs 46.8%, *P* = 0.005) and rated the perceived importance of sexuality as important or very important (75.1% vs 68.6%, *P* = 0.048). These differences represent small effects (Table 2).

**Table 2:** Sexual behaviour, psychosocial factors, and dimensions of sexual self‐concept in the 1:1 propensity score‐matched vasectomy group (*n* = 570) and no vasectomy group (*n* = 570).

Variable	Vasectomy (*n* = 570)	No vasectomy (*n* = 570)	*P*	Effect size
Sexual behaviour				
Partnered sexual activity, % (*n*)				
No	5.6 (32)	11.2 (64)	**<0.001**	0.14
Once a month or less	16.5 (94)	21.3 (121)		
A few times a month to once a week	51.8 (295)	47.5 (271)		
2–3 times a week	21.2 (121)	17.2 (98)		
4 times or more per week	4.9 (28)	2.8 (16)		
Satisfaction with frequency of sex^+^, % (*n*)				
Desire for more frequent sexual activity	57.0 (325)	61.1 (348)	0.373	0.05
Desire for less frequent sexual activity	0.2 (1)	0.4 (2)		
No desire for change	42.8 (244)	38.6 (220)		
Sexual satisfaction, % (*n*)				
(Very) dissatisfied	17.2 (98)	19.5 (111)	**0.005**	0.10
More or less satisfied	26.5 (151)	33.7 (192)		
(Very) satisfied	56.3 (321)	46.8 (267)		
Perceived importance of sexuality, % (*n*)				
(Very) important	75.1 (428)	68.6 (391)	**0.048**	0.07
More or less important	22.5 (128)	27.9 (159)		
(Very) unimportant	2.5 (14)	3.5 (20)		
Number of sexual partners (lifetime), % (*n*)				
1	7.7 (44)	7.4 (42)	0.952	0.02
2–10	61.4 (350)	61.9 (353)		
11–30	23.9 (136)	23.0 (131)		
>30	7.0 (40)	7.7 (44)		
Psychosocial factors				
Depression (PHQ‐2), % (*n*)				
Negative (<3)	96.7 (551)	97.0 (553)	0.735	0.01
Positive (≥3)	3.3 (19)	3.0 (17)		
Anxiety (GAD‐2), % (*n*)				
Negative (<3)	97.7 (557)	96.0 (547)	0.090	0.05
Positive (≥3)	2.3 (13)	4.0 (23)		
QoL score, mean (SD)	79.4 (14.0)	78.4 (14.8)	0.269	0.03
QoL score, % (*n*)				
Lower (<70)	23.7 (135)	25.6 (146)	0.450	0.02
Higher (≥70)	76.3 (435)	74.4 (424)		
Dimensions of sexual self‐concept				
Body image score, mean (SD)	3.9 (0.6)	3.9 (0.6)	0.649	0.01
Toughness score, mean (SD)	2.2 (0.6)	2.3 (0.7)	0.332	0.03
Sexual self‐esteem score, mean (SD)	3.9 (0.5)	3.8 (0.5)	0.149	0.04
Perceived pressure with regard to sexuality score, mean (SD)	1.6 (0.6)	1.6 (0.6)	0.756	0.01

Bold values statistically significant at *P* < 0.05. Education was categorized as low (no secondary school certificate or lower secondary school certificate), intermediate (intermediate secondary school certificate), and high (university entrance qualification or tertiary degree).

**Fig. 2:**
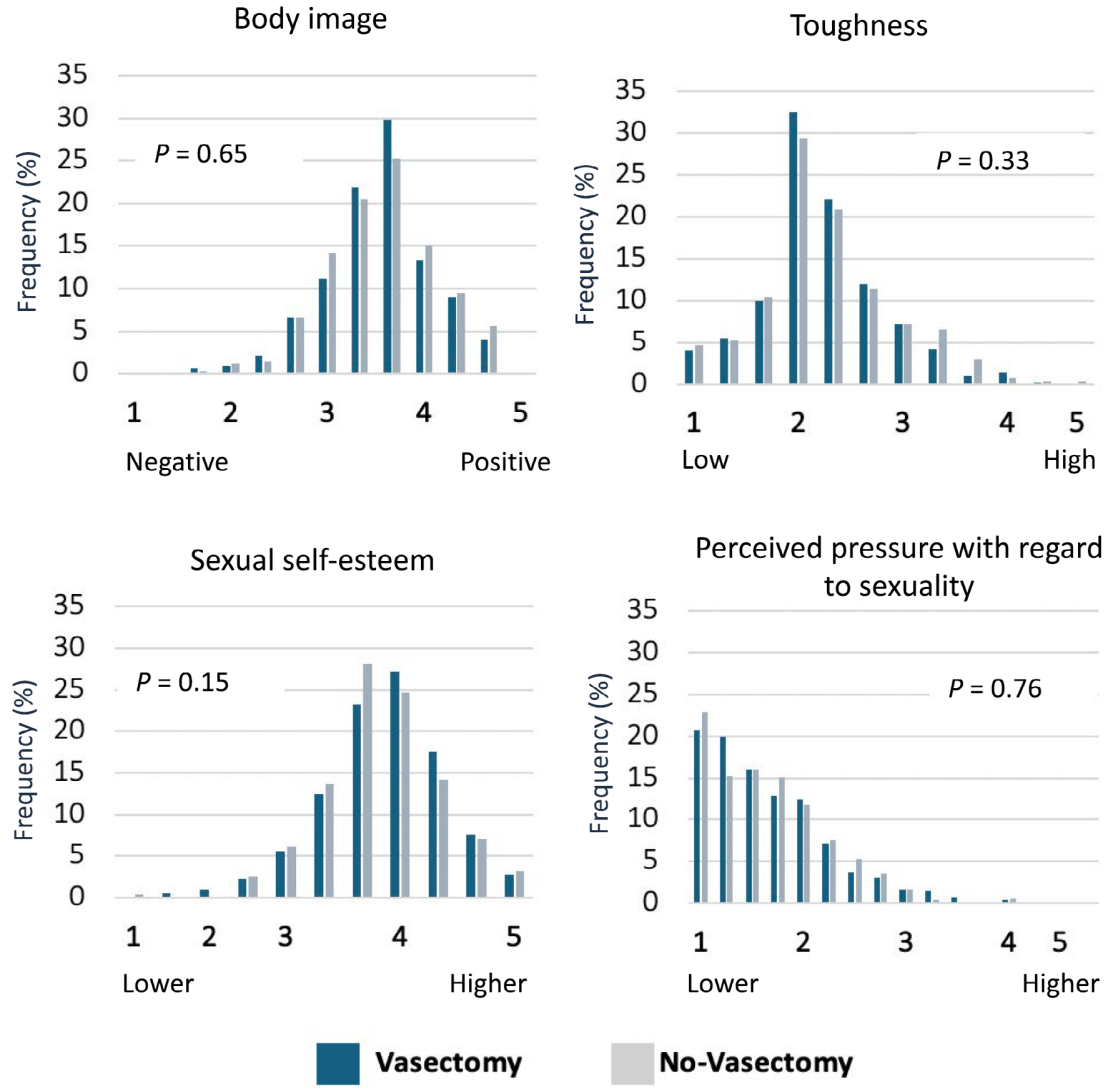
Frequency distribution of the responses on the 5‐point Likert scale for each of the four dimensions of the sexual self‐concept, comparing the vasectomy (blue) and no‐vasectomy (grey) group.

No differences were observed between the two groups in terms of symptoms of depression or anxiety, or level of QoL. When comparing the propensity score‐matched groups regarding the four dimensions of the sexual self‐concept, no differences were found regarding body image, toughness, sexual self‐esteem, and perceived pressure with regard to sexuality (Fig. 2; Table 2).

## Discussion

### Vasectomy and Persistent Myths

Vasectomy, as a safe and effective male contraceptive procedure, continues to face low adoption rates, influenced by persistent myths and misconceptions about its impact on sexual function and physical appearance [1, 2, 6, 7, 9]. These misconceptions wrongly equate vasectomy with castration, suggesting that vasectomy leads to significant changes in physical characteristics, personality, and sexual ability, including loss of libido, ejaculation and potency, therefore portraying vasectomy as a threat to ‘traditional’ masculinity [7]. In general, masculinity refers to gender norms and practices specific to a particular time and context, shaping socially accepted ideals of being a man and how a man should behave. It is not solely influenced by individual feelings but is also shaped by broader social values and beliefs. The influence of social, cultural, and regulatory environments on contraception may limit direct comparisons between the aforementioned studies and the results of this study, but a recent review concluded that also in high‐income countries, many men still perceive vasectomies as a threat to traditional masculinity [6].

### Vasectomy and Dimensions of the Sexual Self‐Concept

In this study of men around the age of 50 years, no differences in sexual self‐concept between men who had undergone a vasectomy ~8 years earlier and men who had not undergone the procedure were found. This suggests that body image or sexual self‐esteem may not be negatively influenced by a vasectomy. Further, toughness as one aspect of the understanding of masculinity and perceived pressure with regard to sexuality did not differ between the two groups. Moreover, men who had undergone a vasectomy were more sexually active, reported greater sexual satisfaction and rated sexuality as more important. While these patterns are suggestive, they should be interpreted cautiously as they may reflect underlying associations rather than direct effects of vasectomy.

These null findings regarding body image, toughness, sexual self‐esteem, and perceived pressure directly challenge the above‐mentioned cultural myths that vasectomy may threaten the traditional understanding of masculinity. Despite societal perceptions, men who underwent vasectomy did not report diminished masculine identity or toughness as selected dimensions of the sexual self‐concept, suggesting that such fears may be largely unfounded. Again, this may suggest that vasectomy does not affect gender‐related and sexual self‐perception.

To the best of our knowledge no study has compared body image between men with or without vasectomy. It is known from female contraceptive methods, such as the birth control pill, that this can lead to a more negative body image or negative weight control behaviour of women. These contraceptives are based on hormonal changes and can impact body composition through these hormonal alterations [29]. It was unclear whether men might also develop a different body image post‐vasectomy due to misconceptions about potential changes in testosterone levels, for example. However, this study found no differences in perception of body image between men with or without a vasectomy.

Another myth regarding vasectomy is its alleged negative impact on sexual function. The presence of sexual function problems is often discussed as being correlated with lower sexual self‐esteem and higher perceived pressure with regard to sexuality [30]. These two aspects are major components of sexual concepts and have an important impact on sexual health. Studies evaluating sexual self‐esteem or perceived pressure with regard to sexuality post‐vasectomy are currently lacking. Another analysis from the BMH Study revealed no differences in the prevalence of erectile dysfunction, ejaculatory problems, or low libido between men around the age of 50 years with and without a vasectomy. Consistent with the absence of differences in sexual problems, in this study, no differences in sexual self‐esteem or perceived pressure with regard to sexuality between men with or without a vasectomy were found.

### Vasectomy and Sexual Behaviour and Psychosocial Outcomes

In terms of sexual satisfaction and frequency of partnered sexual activity, findings from this study align with previous research showing no adverse effects following vasectomy [8, 11]. Furthermore, other studies suggest that vasectomy positively impacts couples’ sexual satisfaction, possibly also due to the elimination of the need for hormonal contraception, with female partners reporting notable improvements in arousal, satisfaction, orgasm, and libido [11]. In this study, vasectomised men had a higher frequency of partnered sexual activity within the last 3 months, consistent with prior research [31]. This may be due to the removal of reproductive concerns or fear of unintended pregnancies for both partners, as previously discussed in other studies [8, 11]. In addition, men who had undergone a vasectomy perceived sexuality as more important compared to men without vasectomy. Actively participating in contraception in a self‐determined and proactive manner may, therefore, also be of greater interest to these men. Taking a more active role in decision‐making in terms of contraception and assuming responsibility by undergoing vasectomy may therefore result in being more sexually satisfied. Also, it is possible that men reporting higher sexual activity or satisfaction may also have more stable partnerships, which could influence their decision to undergo vasectomy. Thus, selection effects related to relationship status or sexual functioning may partly explain observed associations.

In terms of psychosocial parameters, no differences were observed in psychosocial outcomes, including symptoms of depression or anxiety, as well as overall QoL, between men with and without vasectomy. These findings might provide additional reassurance that undergoing vasectomy is not associated with poorer mental health or psychosocial well‐being.

### Social and Cultural Barriers to Vasectomy

Another critical barrier to vasectomy uptake is that contraception is typically framed as a ‘woman's responsibility’. In terms of global gender gaps in healthcare use and outcomes for men, men are often excluded from contraceptive discussions, and healthcare providers predominantly target female partners for counselling. For example, United States insurance data show that only 0.78% of men aged 18–64 years received vasectomy counselling [2]. Expanding contraceptive counselling to actively include men could challenge traditional gender norms that contraception is solely a woman's responsibility and actively involve men in reproductive health decisions and increase vasectomy rates.

### Strengths and Limitations

It is important to emphasise the use of 1:1 propensity score matching in our statistical analysis, which adjusts for potential sociodemographic and lifestyle confounding factors, thereby improving comparability. This approach enhances the ability to control for confounding influences and ensures greater generalisability when evaluating the potential impact of vasectomy on sexual self‐concept, sexual behaviour, and psychosocial parameters. By doing so, the observed differences may more likely be linked to a vasectomy rather than external factors. Unlike previous studies that assessed the effects of vasectomy only a few months after the procedure, this study focused on 50‐year‐old men, with an average of 8 years since undergoing vasectomy, allowing for the evaluation of long‐term effects.

Moreover, some limitations must be acknowledged, such as the absence of longitudinal data on men's sexual self‐concept before and after vasectomy. This limits causal assumptions on the impact of a vasectomy on the assessed parameters. Men who decide to have a vasectomy may have already had a better sexual self‐concept, higher partnered sexual activity, higher sexual satisfaction, and higher perceived importance of their sex life, therefore potentially influencing baseline comparisons. Additionally, despite the use of propensity score matching, residual confounding by unmeasured variables cannot be fully excluded. Potential self‐report biases, including social desirability effects, and selection biases due to study participation may also influence the results. Moreover, it is important to consider the cross‐sectional study design when interpreting the present results. The missing observed differences between men with or without a vasectomy in selected dimensions of the sexual self‐concept of middle‐aged men should be interpreted as associations rather than causal effects. Moreover, some scales assessing sexual self‐concept dimensions comprised only three items and demonstrated slightly below‐threshold internal consistency, which may limit reliability and should be considered when interpreting the findings. But even if these exploratory analyses cannot yet provide confirmatory evidence, they already offer important insights and help to generate hypotheses for future longitudinal research. Future research should therefore prioritise longitudinal studies that assess men both before and after vasectomy to determine potential declines or changes over time, which would provide a more comprehensive understanding of the procedure's impact on dimensions of the sexual self‐concept. Considering the different cultural and social influences that shape the sexual self‐concept of men, it is important to highlight that this study included men living in Bavaria, Germany. Therefore, the observed associations may differ in other cultural contexts.

## Conclusion

In conclusion, this study contradicts common misconceptions about vasectomy. It shows that vasectomy is not associated with detrimental effects on sexual self‐concept, sexual satisfaction and psychological well‐being in the long run. By addressing myths and educating both men and healthcare providers, barriers to vasectomy adoption can be reduced and male participation in family planning can be encouraged as vasectomy offers a safe, effective, and minimally invasive contraceptive option. It allows men to take an active role in their reproductive health.

## Author Contributions

Conceptualisation: Kathleen Herkommer and Andreas Dinkel; Data curation: Lilly J. Schmalbrock, Stefan Schiele, Martina Kron and Helga S. Schulwitz; Formal analysis: Stefan Schiele, Martina Kron, Lilly J. Schmalbrock and Camille Nöckel; Investigation: Lilly J. Schmalbrock, Kathleen Herkommer and Camille Nöckel; Methodology: Kathleen Herkommer, Andreas Dinkel; Project administration: Kathleen Herkommer and Jürgen E. Gschwend; Resources: Kathleen Herkommer and Jürgen E. Gschwend; Software: Stefan Schiele, Martina Kron and Helga S. Schulwitz; Supervision: Kathleen Herkommer and Andreas Dinkel; Validation: Lilly J. Schmalbrock, Valentin H. Meissner, Matthias Jahnen; Visualization: Lilly J. Schmalbrock, Camille Nöckel; Writing – original draft: Lilly J. Schmalbrock and Kathleen Herkommer; Writing – review and editing: Camille Nöckel, Valentin H. Meissner, Andreas Dinkel, Stefan Schiele, Martina Kron, Matthias Jahnen, Helga S. Schulwitz and Jürgen E. Gschwend. All authors have read and agreed to the published version of the manuscript.

## Disclosure of Interests

None declared.

## Funding Statement

No funding.

## Informed Consent Statement

Written informed consent was obtained from all subjects enrolled. The study was approved by the Ethics Committee of the TUM University Hospital (approval number: 699120SSR).

## Work at Conferences

Some parts of the data were presented at the 26th Congress of the European Society for Sexual Medicine (ESSM) in Vienna and at the 40th Annual Congress of the European Association of Urology (EAU25) in Madrid.

## Data Availability

The data presented in this study are available on request from the corresponding author. The data are not publicly available due to data safety reasons.

## References

[1] Dohle GR , Diemer T , Kopa Z , Krausz C , Giwercman A , Jungwirth A . European Association of Urology guidelines on vasectomy. Eur Urol 2012; 61: 159–163 22033172 10.1016/j.eururo.2011.10.001

[2] Velez D , Pagani R , Mima M , Ohlander S . Vasectomy: a guidelines‐based approach to male surgical contraception. Fertil Steril 2021; 115: 1365–1368 33879342 10.1016/j.fertnstert.2021.03.045

[3] Peacock J , James G , Atkinson M , Henderson J . Complications of vasectomy: results from a prospective audit of 105 393 procedures. BJU Int 2024; 134: 789–795 38989696 10.1111/bju.16463

[4] Jacobstein R , Radloff S , Khan F et al. Down but not out: vasectomy is faring poorly almost everywhere‐we can do better to make it a true method option. Glob Health Sci Pract 2023; 11: e2200369 36853640 10.9745/GHSP-D-22-00369 PMC9972380

[5] Shih G , Dubé K , Dehlendorf C . “We never thought of a vasectomy”: a qualitative study of men and women's counselling around sterilization. Contraception 2013; 88: 376–381 23177918 10.1016/j.contraception.2012.10.022

[6] Nicholas L , Newman CE , Botfield JR , Terry G , Bateson D , Aggleton P . Men and masculinities in qualitative research on vasectomy: perpetuation or progress? Health Sociol Rev 2021; 30: 127–142 34018906 10.1080/14461242.2020.1789486

[7] Marván ML , Ehrenzweig Y , Hernández‐Aguilera D . Mexican men's view of vasectomy. Am J Mens Health 2017; 11: 610–617 27339767 10.1177/1557988316655743 PMC5675241

[8] Engl T , Hallmen S , Beecken WD , Rubenwolf P , Gerharz EW , Vallo S . Impact of vasectomy on the sexual satisfaction of couples: experience from a specialized clinic. Cent European J Urol 2017; 70: 275–279 10.5173/ceju.2017.1294 PMC5656365 29104791

[9] Tzelves L , Talyshinskii A , Nedbal C et al. Patient perspectives on vasectomy: findings from a TikTok® content analysis. Int J Impot Res 2024. Epub ahead of print. 10.1038/s41443-024-00931-5 38877106

[10] Smith A , Lyons A , Ferris J , Richters J , Pitts M , Shelley J . Are sexual problems more common in men who have had a vasectomy? A population‐based study of Australian men. J Sex Med 2010; 7: 736–742 19878443 10.1111/j.1743-6109.2009.01565.x

[11] Mohamad Al‐Ali B , Shamloul R , Ramsauer J et al. The effect of vasectomy on the sexual life of couples. J Sex Med 2014; 11: 2239–2242 24820516 10.1111/jsm.12567

[12] Ashley M , Drouin S , Shaughnessy K . Measuring sexual self‐concept: a methodological review. Psychol Sex 2024; 15: 254–277

[13] Pöhlmann K , Roth M , Brähler E , Joraschky P . Der Dresdner Körperbildfragebogen (DKB‐35): validierung auf der Basis einer klinischen stichprobe. Psychother Psychosom Med Psychol 2014; 64: 93–100 23966276 10.1055/s-0033-1351276

[14] Snell W , Papini D . The sexuality scale: an instrument to measure sexual‐esteem, sexual‐depression, and sexual‐preoccupation. J Sex Res 1989; 26: 256–263

[15] Schmalbrock LJ , Schweitzer TMP , Meissner VH et al. Sexual dissatisfaction and associated factors among middle‐aged men—data from the Bavarian Men's Health study. J Sex Med 2025; 22: 1244–1252 40413794 10.1093/jsxmed/qdaf122

[16] Thompson EH , Pleck JH . The structure of male role norms. Am Behav Sci 1986; 29: 531–543

[17] Deutsch AR , Hoffman L , Wilcox BL . Sexual self‐concept: testing a hypothetical model for men and women. J Sex Res 2014; 51: 932–945 23998689 10.1080/00224499.2013.805315

[18] Horenstein D , Houston BK . The effects of vasectomy on postoperative psychological adjustment and self‐concept. J Psychol 1975; 89: 167–173 1151892 10.1080/00223980.1975.9915747

[19] Herkommer K , Meissner VH , Dinkel A et al. Prevalence, lifestyle, and risk factors of erectile dysfunction, premature ejaculation, and low libido in middle‐aged men: first results of the Bavarian Men's Health‐study. Andrology 2024; 12: 801–808 37676020 10.1111/andr.13524

[20] Gil S . Body image, well‐being and sexual satisfaction: a comparison between heterosexual and gay men. Sex Relat Ther 2007; 22: 237–244

[21] Yean C , Benau E , Dakanalis A , Hormes JM , Perone J , Timko A . The relationship of sex and sexual orientation to self‐esteem, body shape satisfaction, and eating disorder symptomatology. Front Psychol 2013; 4: 878 24348441 10.3389/fpsyg.2013.00887 PMC3841718

[22] Hyde Z , Flicker L , Hankey GJ et al. Prevalence of sexual activity and associated factors in men aged 75 to 95 years: a cohort study. Ann Intern Med 2010; 153: 693–702 21135292 10.7326/0003-4819-153-11-201012070-00002

[23] Korfage IJ , Pluijm S , Roobol M , Dohle GR , Schröder FH , Essink‐Bot M‐L . Erectile dysfunction and mental health in a general population of older men. J Sex Med 2009; 6: 505–512 19067789 10.1111/j.1743-6109.2008.01111.x

[24] Löwe B , Wahl I , Rose M et al. A 4‐item measure of depression and anxiety: validation and standardization of the patient health Questionnaire‐4 (PHQ‐4) in the general population. J Affect Disord 2010; 122: 86–95 19616305 10.1016/j.jad.2009.06.019

[25] Scott NW , Fayers P , Aaronson NK et al. EORTC QLQ‐C30 Reference Values Manual, 2nd edn. Madrid, Spain: EORTC Quality of Life Group, 2008

[26] Aaronson NK , Ahmedzai S , Bergman B et al. The European Organization for Research and Treatment of Cancer QLQ‐C30: a quality‐of‐life instrument for use in international clinical trials in oncology. J Natl Cancer Inst 1993; 85: 365–376 8433390 10.1093/jnci/85.5.365

[27] Thiele A , Ottermann C , Degenhardt A . Sensation seeking und aggressivität bei jungen männern. Validierung der “male role norms scale”. Poster presented at the Meeting of the Division Developmental Psychology, German Psychological Society, Potsdam, 2–5 September. 2001

[28] Meissner VH , Söhne VA , Dinkel A et al. Dimensions of sexual self‐concept and sexual dysfunctions in middle‐aged men: results of the Bavarian Men's Health‐study. Int J Impot Res 2025; 38: 430–437 40615569 10.1038/s41443-025-01119-1 PMC13171599

[29] Gillen MM , Rosenbaum DL , Winter VR , Bloomer SA . Hormonal contraceptive use and women's well‐being: links with body image, eating behavior, and sleep. Psychol Health Med 2024; 29: 905–916 37220269 10.1080/13548506.2023.2216468

[30] Özkent MS , Hamarat MB , Taşkapu HH , Kılınç MT , Göger YE , Sönmez MG . Is erectile dysfunction related to self‐esteem and depression? A prospective case–control study. Andrologia 2021; 53: e13910 33215726 10.1111/and.13910

[31] Guo DP , Lamberts RW , Eisenberg ML . Relationship between vasectomy and sexual frequency. J Sex Med 2015; 12: 1905–1910 26272461 10.1111/jsm.12962

